# Experimental Investigation on Pore Structure Characterization of Concrete Exposed to Water and Chlorides

**DOI:** 10.3390/ma7096646

**Published:** 2014-09-16

**Authors:** Jun Liu, Kaifeng Tang, Qiwen Qiu, Dong Pan, Zongru Lei, Feng Xing

**Affiliations:** Guangdong Provincial Key Laboratory of Durability for Marine Civil Engineering, Shenzhen University, Shenzhen 518060, Guangdong, China; E-Mails: liujun@szu.edu.cn (J.L.); tangkaifeng@email.szu.edu.cn (K.T.); 2011090158@email.szu.edu.cn (D.P.); leizongru@email.szu.edu.cn (Z.L.)

**Keywords:** pore structure, concrete, deionised water, chlorides, leaching, fly ash

## Abstract

In this paper, the pore structure characterization of concrete exposed to deionised water and 5% NaCl solution was evaluated using mercury intrusion porosity (MIP), scanning electron microscopy (SEM) and X-ray diffraction (XRD). The effects of calcium leaching, fly ash incorporation, and chloride ions on the evolution of pore structure characteristics were investigated. The results demonstrate that: (i) in ordinary concrete without any fly ash, the leaching effect of the cement products is more evident than the cement hydration effect. From the experimental data, Ca(OH)_2_ is leached considerably with the increase in immersion time. The pore structure of concrete can also be affected by the formation of an oriented structure of water in concrete materials; (ii) incorporation of fly ash makes a difference for the performance of concrete submersed in solutions as the total porosity and the pore connectivity can be lower. Especially when the dosage of fly ash is up to 30%, the pores with the diameter of larger than 100 nm show significant decrease. It demonstrates that the pore properties are improved by fly ash, which enhances the resistance against the calcium leaching; (iii) chlorides have a significant impact on microstructure of concrete materials because of the chemical interactions between the chlorides and cement hydrates.

## 1. Introduction

Pore structure is one of the most important parameters, which determines the properties of cement-based materials. Pore structure is generally characterized by total porosity and pore size distribution (PSD). Previous researches have indicated that chloride ions, originating from deicing salts or sea water, can affect the pore structure characterization of the cement materials [1,2], as the pore network provides the access for some ions to diffuse into the mixture. The permeability of the concrete has a remarkable effect on the structural durability because it determines the rate of aggressive ingress [3,4,5]. Many factors are responsible for determining the size and connectivity of pore structure, including the water-binder ratios, mineral additives and curing condition [6,7,8]. Technically speaking, a concrete with high durability can be produced by introduction of highly effective agent or mineral admixtures. Apart from the concrete itself, exposure to air or marine environment also plays a vitally important part in the development of the pore structure. Aggressive substances like carbon dioxide and chlorides can change the microstructure of concrete due to the physical adhesion and the chemical reactions [9,10,11,12].

Fly ash, a by-product from thermal power plants and metallurgical industrials, has found common practice in civil engineering, in response to the increasing requirement of energy across the world. Many researchers have studied the macroscopic properties (e.g., mechanical properties, workability) of fly ash as well as the micro-mechanisms like transport properties [13,14,15]. Previous researchers [16,17,18,19] have reported that the pozzolanic materials with fine particles can fill in the space enclosed by cement hydrates and thus block the capillary pores of concrete. Therefore, the density of concrete is improved by fly ash inclusion. However, the cement hydrate like Ca(OH)_2_ and C–S–H may be dissolved into water when concrete is exposed to marine environment [20,21,22]. The dissolution can have an adverse impact on the pore structure of concrete.

Even though numerous investigations regarding the performance of pore structure have been carried out [23,24,25,26], there are still fundamental limitations in experiments in determining the direction of effects of deionised water and chloride submersion on pore structure. Although there have been some methods proposed to accelerate and quantify the leaching effect of cement product under marine submersion, such as providing a high temperature, introducing an electrical field, employing ammonium nitrate solutions [27,28], nitric acids [29] or organic acids [30]. These methods are not able to simulate the real life environments that concrete specimens are exposed to. Deionized water can become the candidate for concrete exposure in the experiment as it is capable of proximately simulating the liquid environments containing low ions, such as rain water, ground water, river water or fresh water lake. Additionally, NaCl solution can imitate the marine or coastal environment for the concrete surrounding. The objective of this paper is to study the pore structure characterization of concrete exposed to deionised water as well as NaCl solution. Effects of fly ash inclusion and chloride ions on the development of pore structure were discussed.

## 2. Results and Analysis

### 2.1. The Pore Structure Characterization of Concrete Submersed in Deionised Water

Mercury intrusion porosimetry (MIP) has been widely used to measure the porosity and pore size distribution of cement-based materials for years [31,32,33]. The diameter of pores was calculated according to Washburn equation [34], as described in Equation (1) below:

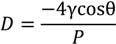
(1)
Where *D* is the pore diameter (μm), γ is the surface tension of mercury (mN/m), θ is the contact angle between mercury and the concrete (°) and *P* is the applied pressure (Mpa). The surface tension of mercury applied here is 480 mN/m, and the contact angle between mercury and concrete used is 117°.

**Figure 1:**
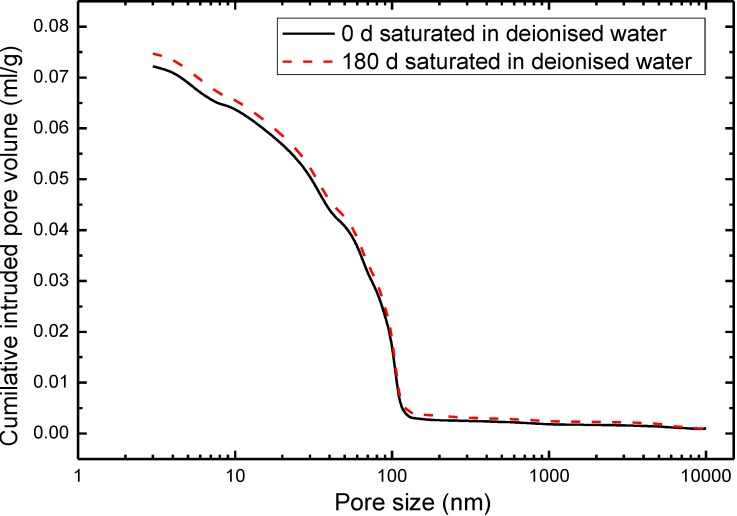
The cumulative intruded pore volume *vs.* pore diameter curves for ordinary Portland cement.

Through Barrett–Joyner–Hanlenda (BJH) interpretation [35], the pore diameter distribution of ordinary portland cement (OPC) at 0 day and 180 days of saturation in deionised water is exhibited in Figure 1.

As can be seen from the data in Figure 1, the mixtures OPC of 180 days immersion of deionised water showed higher cumulative intruded pore volume as compared to 0 day immersion. It should be noted that cement-based materials are considered as porous media which consist of different types of pores including space between the C–S–H gels, capillary pores and void pores. As for porous solids, internal moisture movements and structural transformations of water, such as the formation of an ordered structure of water, freezing of water into ice, are known to cause disruptive volume changes of many types [36]. Formation of structure of the oriented water molecules in micropores is known to cause expansion in many systems. Given that the pore diameter of concrete covers a wide length scale from the nanoscale to microscale, the surface tension of pores is so high (due to the large surface area or energy) that the hydrogen bond of bulk water is destroyed and the water molecules are oriented to an ordered strucuture. This oriented water, being less dense than the bulk water, will require more space and will therefore tend to cause expansion [36].

Furthermore, the result of Figure 1 can be explained via a mechanism of progressive leaching of calcium. Deionised water in contact with cement-based materials creates concentration gradients that lead to the diffusion of ions contained in the interstitial solution [20]. The progressive dissolution of solid Portlandite is therefore occurs due to the modification of chemical balances. The ongoing decrease in Ca/Si ratio in the solids results in disability of C–S–H gel and causes its decalcification [37,38]. In the leaching procedure, the porosity increases and the microstructure is modified, leading to an increase in permeability and a decrease in bulk density [39]. Christophe Carde *et al.* [40] investigated the leaching of Ca(OH)_2_ and a progressive decalcification of C–S–H with the use of a 50% concentrate solution of ammonium nitrate, and established a model of both strength decreasing and porosity increasing during the leaching process. As the dissolution of Ca(OH)_2_ increases to 25%, the micro pore structure of concrete changes dramatically, from a dense mixture (low pore volume: 49.09 × 10^−2^ mL and small pore size: 7.5 nm) to an incompact mixture (high pore volume: 69 × 10^−2^ mL and large pore size: l00 nm).

**Figure 2:**
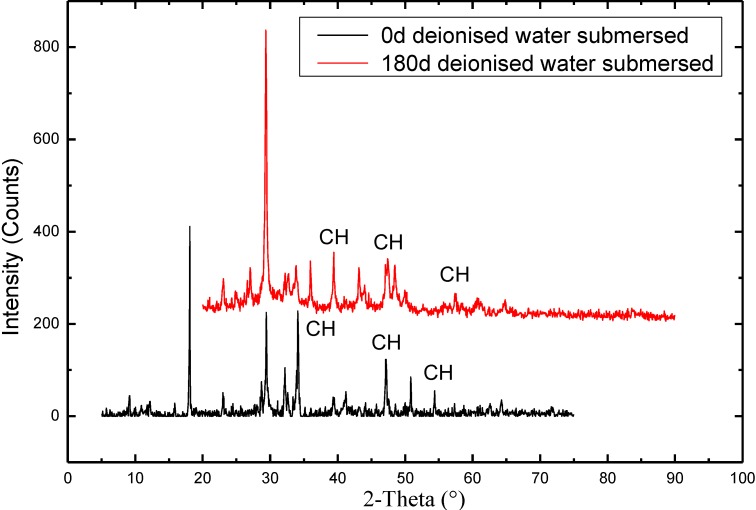
X-ray diffraction (XRD) patterns of Ordinary Portland Cement (OPC) at the submersion ages of 0 and 180 days.

In this experiment, the results of X-ray diffraction data of OPC at the immersion ages of 0 and 180 days are presented in Figure 2. The characteristic broad peaks of Ca(OH)_2_ are recorded as 34°, 47°, 51° and 64°, respectively. Based on the results of characteristic broad peak, as compared to the neat concrete of 0 day immersion time, the peak size of Ca(OH)_2_ of 180 days pure water submersion is evidently lower, which indicates the depletion of Ca(OH)_2_. The density and the pore structure of concrete is thought to be changed undesirably, along with the ongoing consumption of Ca(OH)_2_. With the increasing the immersion time, the pore structure of the concrete deteriorates.

### 2.2. Effects of Fly Ash on the Pore Structure of Concrete

**Figure 3:**
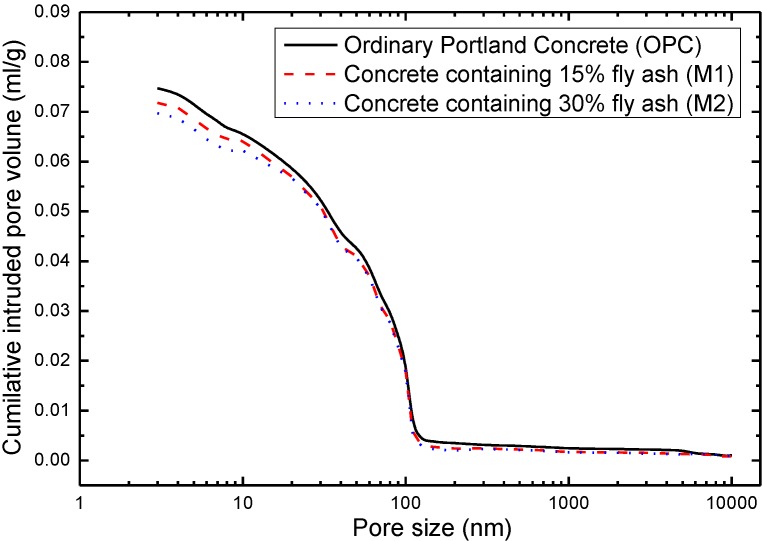
Cumulative mercury porosimetry of all specimens exposed to deionised water.

Figure 3 depicts the cumulative intruded pore volume of concrete incorporating 0%, 15% and 30% fly ash, at the age of 180 days exposure to deionised water. It can be found that the mixture containing fly ash replacement shows lower cumulative intruded pore volume than the control mixture. The intrusion is more pronounced with the increase in the amount of fly ash substitution. The observation is firstly related to the double effects of fly-ash substitution of concrete. Physically, fly ash can serves as the micro-filler that fills in the space among the calcium silicate hydrate (CSH) gels, which blocks the capillary pores and hence helps to achieve a denser concrete. From the chemical perspective, fly ash with Portlandite (Ca(OH)_2_) and water generates hydration products similar to those of Portland cement, * i.e.*, calcium silicate hydrates (C–S–H), a rigid gel composed of extremely small particles with a layer structure [41]. This reaction is generally called the secondary hydration (pozzolanic reaction). The products C–S–H gels progressively fill in the already formed micro pores of concrete, which results in a higher density level for the concrete. On the other hand, the contact with pure water causes concentration gradients of calcium between the interstitial solution and the outside environment of cementitious materials, as a result of which calcium ions in the pore solution are extracted from the cementitious binder [39]. The initial thermodynamic equilibrium between the pore solution and the solid hydrates is also disturbed by the concentration gradients, which leads to dissolution of Ca(OH)_2_ as well as the decalcification of C–S–H gels. In this regard, the leaching effect may deteriorate the pore structure of concrete. However, as can be seen from the Figure 3, the fly-ash effects have a more pronounced impact on the evolution of the pore structure. Although the leaching can exert an adverse influence on the microstructure of concrete, it needs a stable water permeability to maintain the depletion of cement hydrate. As long as the cementitious material has low porosity and compact pore structure, the water permeability coefficient is too low to maintain the process of dissolution of Ca(OH)_2_ and the subsequent decalcification of C–S–H gels. It should be noted that the porosity of concrete can be lower if the initial Ca(OH)_2_ content in concrete is lower. Consequently, the concrete with a certain amount of fly ash added reflects better leaching resistance than the ordinary concrete. It is therefore to say that the fly ash concrete has a good resistance against calcium leaching, which was also confirmed by previous surveys that have tested the influence of fly ash intrusion on the permeability of concrete. Kunhe Fand *et al.* [42] has reported the late-age properties of roller-compacted concrete with low cement content and high fly ash content. It demonstrates that after a period of water submersion, the Ca^2+^ content in pore solution is lower than that of tap water, which indicates that the dissolution resisting characteristic of the roller-compacted concrete with low cement content and high fly ash content is strong.

As a porous media, cement and concrete materials adopt a pore structure with broad pore size distribution from nanometer to micrometer scales [43]. Generally speaking, the pores of concrete are classified into four major types: gel pores (diameter < 10 nm), transitional pores (diameter 10–100 nm), capillary pores (diameter, 100–1000 nm) and macropores (diameter > 1000 nm) [44]. Pores with different diameters have different influence on concrete. According to the studies outlined in [30,45,46], pores whose diameter of 100 nm is defined as threshold, since the size of the pores are assumed to be detrimental to durability for cement and concrete composites. In this research, the pore sizes of 3–100 nm, 100–200 nm, >200 nm are intended to be discussed.

**Figure 4:**
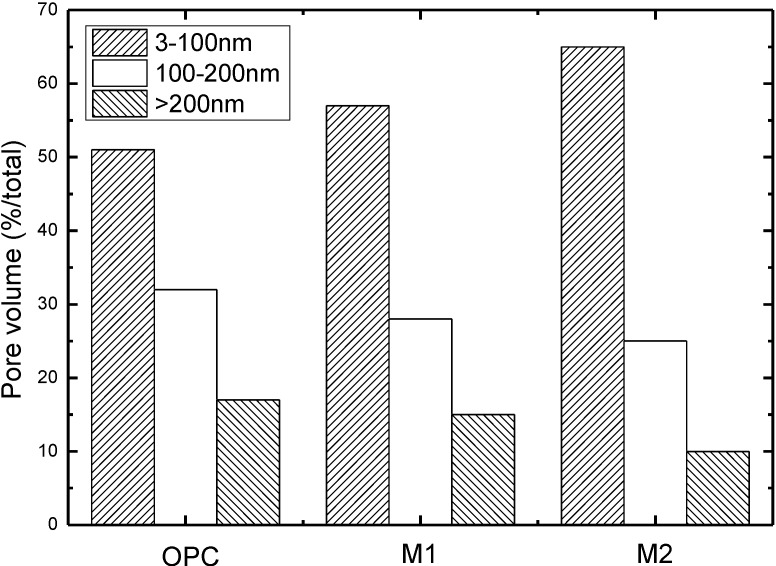
Pore size distribution of concrete immersed in deionised water for 180 days.

**Figure 5:**
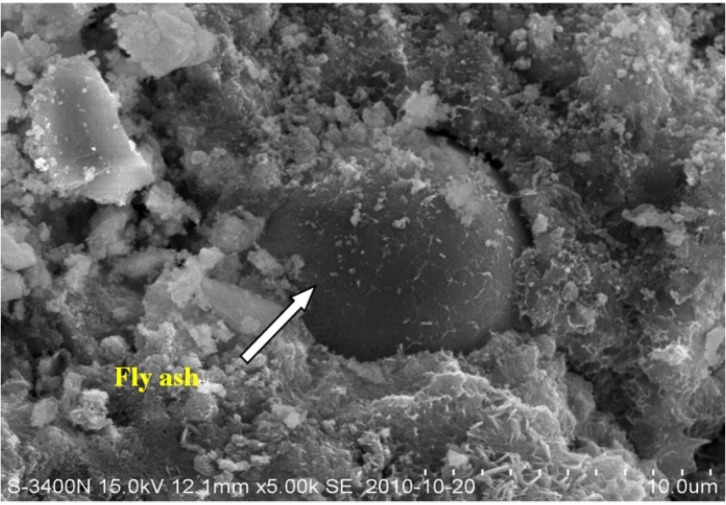
Scanning electron microscopy (SEM) picture of fly ash concrete M1.

Figure 4 illustrates the pore size distribution of concrete immersed in pure water for 180 days. As observed, with respect to the pores in the size range from 3 to 100 nm, M1 and M2 is, 12% and 27% respectively, more than that of OPC. Regarding the pores in the size range of 100–200 nm, M1 and M2 is, 13% and 22% respectively, less than the OPC; As to pores (diameter > 200 nm), M1 and M2 is, 12% and 41%, respectively, less than OPC. It can be concluded that inclusion of fly ash leads to pore structure densification of concrete. Especially when the dosage of fly ash is up to 30%, the pores that is larger than 100 nm significantly declines. It is attributed to that fly ash acts as a pore-filler which reduces the capillary pores (see Figure 5). Another explanation for the phenomenon lies in the secondary hydration of fly ash. At the long time of immersion period, pozzolanic reaction of fly ash occurs due to the dissolution of vitreous gel structure and leaching of Si^4+^ and Al^3+^. Therefore, secondary hydration is therefore to take place to form calcium silicate hydrate (Cao·SO_2_·H_2_O). Therefore, the pore system shows less connectivity, which will lead to very low transport rates.

### 2.3. Effect of Chloride Ingress on the Pore Structure of Concrete

**Figure 6:**
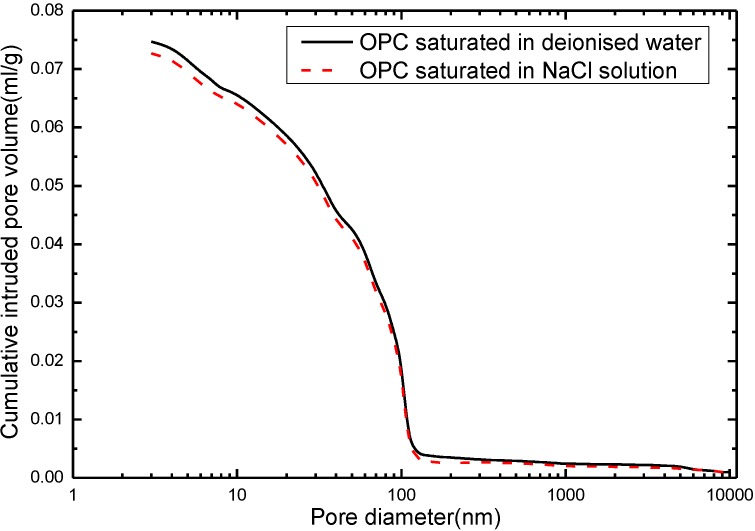
The cumulative intruded pore volume of ordinary Portland concrete immersed with different solutions (deionised water and NaCl solution).

After the chloride ingress, some chlorides can be attached to the pore walls or cement products and react with them, which is called chloride binding [47,48,49]. The chloride binding is commonly classified as the physical binding and the chemical binding. Physical binding occurs when chloride ions transport through the C–S–H type gel surface, and occurs due to electrostatic or Van der Waals forces between chloride and the gel [50]. Chemical binding can be defined as chloride ions interact with C–S–H gel by several mechanisms such as chemisorption into the C–S–H layers, in C–S–H spaces, or becoming bound in the lattice or ion exchange sites of C–S–H gel [51]. These bound chloride ions can give rise to an altered matrix microstructure. The curves in Figure 6 exhibit the cumulative intruded pore volume of the specimens respectively immersed in deionised water and NaCl solution for 180 days. In contrast with the result of deionised water, the pore size of the NaCl solution submersion changes dramatically. The pore size of concrete under chloride saturation has experienced a huge reduction. Similar results were reported by other research [52] that studies the influence of marine sand on the properties of concrete, demonstrating that incorporation of the chlorides can improve the impermeability of concrete.

**Figure 7:**
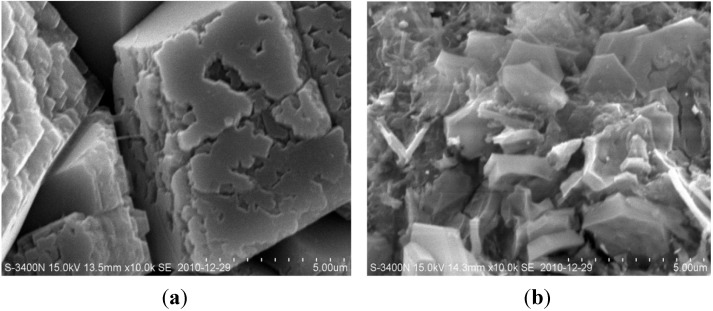
Comparison of scanning electron microscopy (SEM) images of the specimens immersed with different solutions: (**a**) Saturated in deionised water; (**b**) Saturated in NaCl solution.

The chemically bound chlorides in the cementitious materials at the beginning react with the main ordinary Portland cement component, tricalcium aluminate (C_3_A). The reaction product is known as calcium chloroaluminate hydrate, Friedel’s salts, 3CaO·Al_2_O_3_·CaCl_2_·10H_2_O [53], which is the most abundant compound in the material. Tetracalcium aluminoferrite, C_4_AF, also reacts with chloride originating the equivalent ferric salt, 3CaO·Fe_2_O_3_·CaCl_2_·10H_2_O, but the reaction takes place at a slower rate [54,55,56]. The Friedel’s may further react with the Ca(OH)_2_ and form the CaCl_2_ complex, which makes the concrete more compact. As soon as the reaction begins, the binding between the chloride ion and cement product can modify the shape of concrete microstructure. Midgley *et al.* [57] used the NaCl solution as the Cl^−^ source to study the pore size distribution of cement paste under chloride ingress. It was found that the intrusion of chloride ion leads to more fine pores and less big pores. Jensen *et al.* [58,59] had found the same results that ingress of chlorides can produce the solid phase Friedel’s salt at a larger size that precipitates in the pores. As a consequence, the porosity as well as the permeability of the concrete is reduced. Díaz *et al.* [60] use the physical and electrochemical techniques EIS (Electrochemical Impedance Spectroscopy) to quantify microstructural changes of mortar samples saturated with different solutions (deionised water, 0.5 M NaCl and 1 M NaCl). It shows that the presence of Friedel’s salt formed by chlorides distorts the pores and develops the small sized pores (around 10 nm). Figure 7 reveals the SEM image of concrete respectively saturated in deionised water and NaCl solution. It can be observed that the surface of the cement product under saturation of deionised water is evidently rough (Figure 7a), which contains the small pores and denudation. However, the chloride saturation might lead to different observations. Figure 7b shows a smooth surface and dense structure of the interior of concrete. The result is consistent with the findings from the previous studies [61], which suggested that NaCl deicer could chemically react with some of the cement hydrates and form new products in the concrete matrix.

During the long period of immersion in deionised water, the CaO in the cement is dissolved, which results in instability of the cement components and increase in the porosity. This consequence adds fuel to the consumption of Ca(OH)_2_. The more connective pores are in concrete, the more significant is the ion penetration. In terms of submersion in NaCl solution, the process of the cement hydration speeds up, the pore properties in the cement materials are improved, the pore size is smaller, and porosity is lowered. From the results in Figure 6, after 180 days submersion, as to the pores with a diameter larger than 100 nm, samples of NaCl solution is less than that of deionised water. The impermeability is improved by chloride saturation and further slows down the calcium leaching.

## 3. Materials and Methods

### 3.1. Materials

In experimental studies, the P.O 42.5 Portland cement which is produced by Starfish Onoda cement limited company of Shenzhen was used. Type I fly ash (FA) was obtained from a power station in Ma Bay of Shenzhen. The chemical compositions of cement and fly ash are given in Table 1. River sand was employed as the fine aggregate, whose fineness modulus is 2.61 and unit weight is 2632 kg/m^3^. Coarse aggregate that originated from quarry of AnTuo Mountain, Shenzhen, was used. With the unit weight of 2700 kg/m^3^, the maximum and the minimum particle size of coarse aggregate are 20 mm and 5 mm, respectively. Sodium chloride that has purity above 99% was used to perform chloride bath for the specimens. Distilled water was used in concrete production in order to eliminate the influence of other unwelcome ions in water.

**Table 1:** Chemical compositions of cement and fly ash.

Composition (Mass % as Oxide)	Cement	Fly Ash (FA)
Calcium oxide (CaO)	64.67	4.74
Silica (SiO_2_)	18.59	62.32
Alumina (Al_2_O_3_)	4.62	23.95
Iron Oxide (Fe_2_O_3_)	4.17	1.33
Magnesium oxide (MgO)	2.35	2.04
Sulfer trioxide (SO_3_)	3.32	1.25
Potassium oxide (K_2_O)	0.92	0.76
Loss on ignition (LOI)	1.03	3.12

### 3.2. Mix Proportions of Concrete

Water/binder ratio (W/B) was kept constant as 0.47, concrete without any fly ash was taken as the control mixture (Ordinary Portland Cement), while the remaining mixtures had two fly ash replacements with the proportion of 15% and 30%, respectively. The physical characterizations and mix proportions of all materials are summarized in Table 2.

**Table 2:** The physical characterizations and mix proportions of all materials.

Sample	W/B	Compressive Strength	Composition (kg/m^3^)				
			Cement	Sand	Crush Stone	Fly Ash	Water
OPC	0.47	45.4	409	720	1079	0	192
M1	0.47	44.4	348	697	1054	61	192
M2	0.47	36.7	286	689	1041	123	192

OPC means the Ordinary Portland Cement.

### 3.3. Production of Sample and Testing

All specimens with a size of 100 mm × 100 mm × 100 mm were prepared and cured for 28 days under continuous air curing conditions till the submersion testing. The specimen was at first cut into several blocks by a sawing machine. Then, the blocks were cut into fragments with a diameter of not more than 10 mm and with a height of not more than 30 mm, using a small diamond saw. After clearing away the stones and sands at the surface area, the specimens were submersed in deionised water and 5% (by weight of solution) NaCl solution, respectively. The frequency of renewal of the NaCl solution is around one month. The submersion age was set as 90 and 180 days.

### 3.4. Tests for Pore Structure

The specimens were immersed in absolute ethyl alcohol for more than a week in order to stop the cement hydration process. Afterwards, the specimens were put into an oven to be dried under the temperature of 60 °C. In this research, the pore size distribution of concrete was examined by Micromeritics AutoPore IV 9500 (Micromeritics, GA, USA) capable of generating pressure range from subambient to 33,000 pounds. Values of intrusion volume were measured at stepwise increasing pressures equilibrating at each pressure step [62]. Thus, the pore volume can be accessed.

Powder X-ray diffraction (XRD) was employed for calcium hydroxide identification. Scanning Electron Microscopy (SEM) was performed in microscopes. Prior to imaging, the concrete specimens were first crushed into small pieces. These pieces were then placed in a vacuum under 50 °C until constant weight, and coated with a thin gold layer.

## 4. Conclusions

This study discusses an experimental program carried out to investigate the pore structure characterization of cement paste exposed to deionised water and chlorides. The following conclusions can be drawn according to the results of this study:

- As to the ordinary Portland concrete, the deteriorated effect of calcium leaching is found to be the dominant parameter governing the evolution of pore structure in spite of the growth of solid hydration products. During the process of immersion, the leaching phenomenon of Ca(OH)_2_ is obvious. The formation of an oriented structure of water in concrete exposed to water saturation as well bears the responsibility for destruction of the pore structure of concrete.
- The concrete with a certain amount of fly ash added reflects higher calcium leaching resistance degree than the ordinary Portland concrete, which is closely linked to the double effects of fly-ash substitution of concrete: micro-filler effect and pozzolanic reaction. Especially when the dosage of fly ash is up to 30%, the pores that are larger than 100 nm significantly decline. The pore system of fly ash concrete shows less connectivity, which can resist the leaching of Ca(OH)_2_ and dissolution of C–S–H gel.
- The chloride ions can have a significant influence on the microstructure of cement materials. The pore size of concrete under chloride saturation has experienced a huge reduction compared to the deionised water immersion. The SEM images shows that the concrete under chloride saturation has a more smooth surface and denser structure in contrast with the deionised water immersion.
